# Evaluation of Phosphorus Efficiency in Cultivated and Wild Potato Genotypes

**DOI:** 10.3390/plants14243776

**Published:** 2025-12-11

**Authors:** Mousumi Hazarika, Tahar Ashfaq, Klaus J. Dehmer, Silvia Bachmann-Pfabe

**Affiliations:** 1 Satellite Collections North, Gross Luesewitz Potato Collections (GLKS), Genebank Department, Leibniz Institute of Plant Genetics and Crop Plant Research (IPK), Parkweg 3a, 18190 Sanitz, Germany; hazarika@ipk-gatersleben.de (M.H.); mianttahir@gmail.com (T.A.); pfabe@hs-nb.de (S.B.-P.); 2 Plant Nutrition and Soil Science, Faculty for Agriculture and Food Sciences, University of Applied Sciences Neubrandenburg, Brodaer Straße 2, 17033 Neubrandenburg, Germany

**Keywords:** phosphorus efficiency, greenhouse screening, genetic resources, wild *Solanum* accessions

## Abstract

Phosphorus (P) deficiency is a critical constraint to cultivated potato (*Solanum tuberosum* L.) production, while wild potato germplasm is known to harbor traits that enhance tolerance to low P conditions. The aim of this study was to evaluate the tolerance to P deficiency in cultivated and wild potato accessions from a genebank to identify interesting germplasm for potato breeding. Therefore, ten wild potato accessions and 30 cultivated varieties were evaluated under high (15 mg L^−1^) and low (3 mg L^−1^) P regimes for various morphological, physiological, and nutrient uptake traits. Significant genotypic variation was observed across all traits, with notable interspecific differences. While low P generally reduced biomass, several genotypes, particularly *S. stenotomum* accessions, showed enhanced root growth and greater root length per unit shoot biomass. Wild accessions (GLKS 38153, GLKS 38159, GLKS 38161, and GLKS 38163; *S. chacoense*), maintained biomass through efficient internal P use, whereas others displayed high P uptake with limited growth conversion. GLKS 38159 demonstrated remarkable P efficiency, achieving high biomass with lower P uptake. Cultivated varieties, including Ikar, Tiger, Tarzan, Borka, and Fransen, displayed diverse adaptive strategies, including longer roots and sustained biomass. These findings underscore the resilience of wild potatoes to nutrient stress and provide valuable insights for breeders targeting improved P use efficiency in potato varieties.

## 1. Introduction

The increasing unpredictability of weather due to climate change and the rising frequency of extreme climatic events underscore the urgent need for climate-smart, nutrient-efficient crop varieties to ensure global food security [1]. Being a staple food for over 2 billion people and the most important non-grain food crop, potatoes play a crucial role in global food security and stabilizing food prices, and sustaining livelihoods amid these challenges [2,3]. The potato is the fifth most important crop after sugarcane, maize, rice, and wheat, with an annual production of 360 million tons globally [4]. Potato tubers serve not only as an excellent source of carbohydrates with significant nutritional value, but also as a major source of starch for various industrial uses [5].

Along with other essential macronutrients, phosphorus (P) plays a critical role in ensuring the optimal growth and productivity of potatoes [6,7]. It has a comparatively high P requirement, i.e., it can be as high as >400 kg P_2_O_5_ ha^−1^, which is higher than many other crops [8]. Thereby, a considerable yield loss was seen when grown on P-deficient soils [9,10,11]. According to fertilizer recommendations by the Ministry of Agriculture, Fisheries, and Food, United Kingdom (MAFF, presently known as the Department for Environment, Food and Rural Affairs, DEFRA), the P fertilizer requirement of the crop is nearly twice that of wheat and barley, and one-third higher compared to most vegetable crops [12,13]. Thus, an adequate amount of P should be available even at an early growth stage [8,14] to ensure high tuber yield with desirable qualities like swelling power, viscosity, gelatinization temperature, etc. [15,16]. Several studies have reported that P deficiency significantly decreases tuber yield, quality, and size [8].

Although P is vital for crop nutrition, it remains largely unavailable to plants in most soil types. Plants take up P from the soil via inorganic compounds such as orthophosphate (HPO_4_^−^ and H_2_PO_4_^−^) [17,18]. Even when mineral fertilizers are applied, the rapid immobilization of P in soil results in the formation of stable complexes with calcium, aluminum, iron cations, and clay minerals [19,20,21], making P unavailable to the plants. Additionally, depending on the soil type, up to 90% of the total soil phosphorus can exist in organic forms that are less accessible to plants [22]. Furthermore, cultivated potato varieties (*Solanum tuberosum*) typically possess a shallow, sparsely branched root system, restricting their ability to explore a large soil volume for nutrient acquisition [7].

To compensate for this limitation, a high dosage of P fertilization is very often applied [8]. However, such an excessive use of fertilizer leads to low P use efficiency in the crop, along with various serious environmental consequences like eutrophication, contamination of groundwater, and accelerated depletion of the finite mineral P reserves [23].

Over time, plants have developed several adaptive mechanisms to enhance P uptake efficiency and mitigate stress caused by low P availability [24,25]. Morphologically, plants often modify their root-system architecture under P stress by increasing root length, enhancing root-to-shoot ratio, and expanding root surface area, along with root density and lateral root growth [26,27]. Several plant families even produce cluster roots to provide the benefit of increased root surface area and facilitate the mobilization of otherwise unavailable P under low P conditions [6,27]. Biochemically, plants exude organic acids and phosphatases that mobilize bound Pi and promote beneficial rhizosphere microbes [28,29]. In addition, several phosphate-solubilizing microorganisms (PSMs), such as phosphate-solubilizing bacteria (PSB), fungi, and actinomycetes, inhabit the rhizosphere environment of the plants’ roots and are capable of converting insoluble soil P into plant-available forms [6,30]. At the molecular level, enhanced P transporters and Pi starvation-responsive genes were seen to be upregulated to optimize P uptake [25,31].

In summary, an efficient uptake and utilization of the available P has become increasingly important for sustainable crop production. In this context, plant breeding plays a crucial role in developing P-efficient cultivars. For crops like potatoes, which are highly responsive to P availability, it is essential to evaluate how diverse accessions respond to P deficiency, as this enables the identification of genotypes with superior P acquisition and utilization capacities. Genebank collections provide a valuable reservoir of genetic diversity that can be exploited to discover novel traits and alleles associated with enhanced P efficiency. Such efforts are fundamental for modern breeding programs aiming to produce nutrient-efficient varieties capable of maintaining productivity under P-limited conditions [23].

One of the major approaches to identifying P deficiency-tolerant genotypes and further development of resilient cultivars is the exploration of the genetic diversity within wild relatives of modern cultivated potato genotypes, besides exploring the different genotypes of the same species. Wild relatives of cultivated potatoes play a crucial role in breeding programs due to their extensive genetic diversity and adaptation to diverse environmental conditions [32,33]. The genus *Solanum* L., which comprises over 1000 species [34], is widely distributed across the Americas, with members of the tuber-bearing sect. *Petota* Dumort. thriving from the southwestern USA to southern Chile and Argentina [35]. These species have evolved in a range of climatic conditions, making them valuable sources for, e.g., improving P efficiency, biotic and abiotic stress resistance, and overall adaptability in cultivated potatoes. Wild potato species, such as *S. bulbocastanum* Dunal, have been shown to associate with P-solubilizing bacteria (PSB) in their rhizosphere, promoting enhanced P uptake and plant growth [36]. Unlike domesticated crops, which rely heavily on (synthetic) fertilizers, wild relatives often inhabit environments with low-nutrient soils and have developed mechanisms for more efficient nutrient acquisition [37,38]. They, e.g., modulate their rhizosphere microbiota, fostering beneficial microbial communities that facilitate nutrient availability [36,39,40].

The use of wild germplasm has also been helpful in improving resistance to biotic and abiotic stresses in cultivated potatoes. Exotic *Solanum* species harbor valuable genes that enhance disease resistance, stress tolerance, and yield-related traits [41]. For example, introgressions from wild species have successfully conferred disease resistance [42], improved tuber quality [35], and enhanced stress tolerance in tetraploid potatoes [43]. Breeders and researchers continuously explore the secondary gene pool to identify resistant species that can be directly utilized or hybridized with cultivated potatoes [44]. Conventional breeding has played a significant role in developing improved potato varieties, but recent advancements in genomics and sequencing technologies, as highlighted by Tiwari et al., 2022, have accelerated potato improvement through efficient identification and introgression of desirable alleles from wild relatives of *S. tuberosum* [45]. There has been considerable research on the broader potential of wild potato germplasm, including its contributions to resistance against biotic and abiotic stresses.

In terms of P efficiency, Sandaña (2016) [46] assessed various P efficiency and related traits in 22 cultivated potatoes under varying P conditions and reported that P uptake efficiency was more important than utilization efficiency in determining P use efficiency in cultivated accessions. Moreover, they highlighted the significant genotypic variability in these traits, which could be exploited to further improve P efficiency in potato breeding. Previous studies by Wacker-Fester et al. (2019) showed that potato varieties differ in their efficiency to use P from soils low in plant-available P [47]. Similarly, Chea et al. (2021) reported significant variation in potato responses to P deficiency, with some cultivars identified as efficient with higher P uptake efficiency, higher biomass, tuber yield, and P use efficiency under low P [9]. In 2024, Chea et al. further evaluated the morphological and physiological responses, including root system alterations and P use efficiency, of two cultivated potato genotypes to varying P levels, and illustrated that higher P efficiency under low P conditions was coupled with enhanced root growth, P uptake, and translocation [7]. Kirchgesser et al. (2023) studied the root system of 200 potato genotypes under high and low P supply and reported a huge variation in cultivated potato root system architecture and different adaptation strategies to P deficiency [48].

However, less attention has been paid to wild potato germplasm, although it has been proven to carry important traits for cultivar improvement. A screening pot experiment conducted by Balemi in 2011 on 20 potato genotypes (twelve cultivated and eight wild accessions) showed that there is considerably higher variation in P concentration among the wild genotypes compared to cultivated ones under the same P conditions. Specifically, one *S. chacoense* accession and one wild accession of unknown species were identified as promising candidates for improving P efficiency in cultivars [49].

Hence, screening of germplasm collections and studying inheritance patterns remain vital for leveraging wild species to develop resilient, high-performing potato cultivars [50]. Thus, the utilization of wild potato germplasm presents a promising strategy for sustainable crop improvement and long-term food security.

Given these challenges, our study aims to evaluate a collection of 40 *Solanum* accessions belonging to both wild (ten) and cultivated (thirty) species with the goal of investigating the inter- and intraspecific variation among the accessions in their morphological and physiological response to P deficiency conditions and to identify genotypes with higher P efficiency.

## 2. Results

### 2.1. Morphological and Physiological Response of Potato Genotypes to Low P Conditions at the Early Vegetative Stage

**Figure 1:**
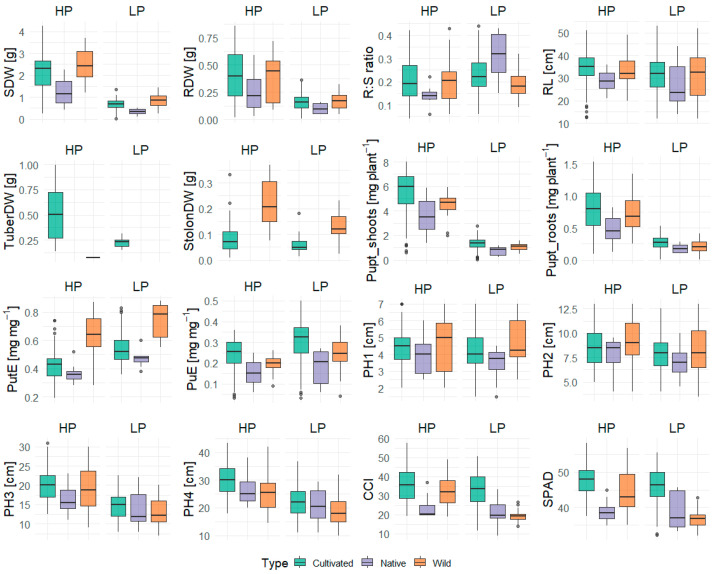
Comparative boxplots of various traits measured under high (HP) and low (LP) phosphorus conditions: shoot dry weight (SDW), root dry weight (RDW), root–shoot ratio (R:S ratio), root length (RL), tuber dry weight (TuberDW), stolon dry weight (StolonDW), P uptake in shoots (Pupt_shoots) and roots (Pupt_roots), P utilization efficiency (PutE), P uptake efficiency (PuE), plant heights over four weeks (PH1–PH4), chlorophyll content index (CCI), and SPAD.

**Figure 2:**
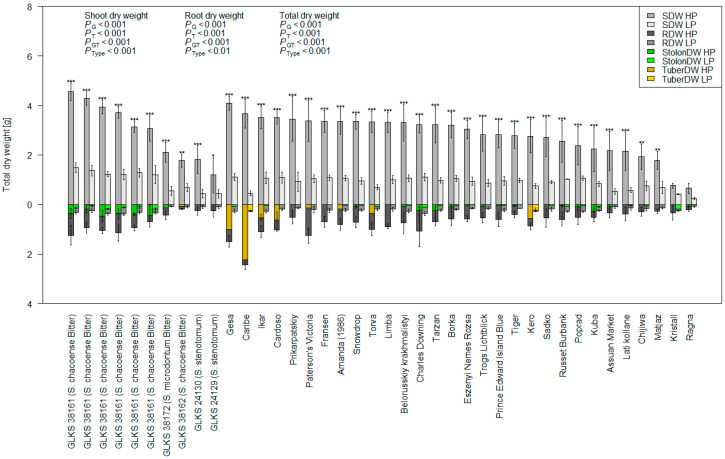
Shoot dry weight (SDW), root dry weight (RDW), stolon dry weight—if produced (StolonDW), tuber dry weight—if produced (TuberDW), and total dry weight (TotalDW) of 40 potato genotypes of the IPK Gene Bank were measured under HP and LP conditions; *p*-values represent the ANOVA results for SDW, RDW, and TotalDW for genotype (G), treatment (T), genotype x treatment (GT), and type/species (Type); asterisks above each bar represent the significant differences in total dry weight (TotalDW) between HP and LP within the same genotype, based on the Tukey test at *p* ≤ 0.05 (*), *p* ≤ 0.01 (**) and *p* ≤ 0.001 (***), respectively.

A summary of the trait values under HP and LP conditions is presented in Table A1 and Table 1. Phenotypic variation in the traits across the cultivated, native, and wild accession groups under both P regimes is represented in Figure 1. The data revealed considerable genotypic variation in their trait response, both within and between the treatments (Figure 1, Table 1). The comparative evaluation of cultivated, native, and wild species under HP and LP conditions revealed significant differences in growth and nutrient-use traits between the species (Table 1, Figure 1). Wild species demonstrated the greatest biomass accumulation, exhibiting significantly higher shoot, root, and total dry weights than both cultivated and native species under both nutrient regimes (Figure 1). Generally, the genotypes showed higher growth under HP conditions, while biomass production was significantly reduced under LP conditions (Figure 1). Shoot dry biomass reduction was lowest for GLKS 38163 (−55.84%, *S. chacoense*, abbreviation *chc*) and highest for variety Caribe (−85.67%) (Figure 2, Table S1). Variety Caribe also showed the largest reduction in root biomass (−88.13%), while the wild type GLKS 38162 (*chc*) exhibited the lowest reduction in root biomass (−24.12%) (Figure 2, Table S1). The TotalDW ranged from 5.253 g per pot (GLKS 38161, *chc*) to 0.272 g per pot (Ragna) under HP and was reduced significantly (*p* ≤ 0.001) to 1.816 g per pot (GLKS 38161, *chc*) to 0.095 g per pot (Ragna) under LP conditions (Table 1, Figure 2), with significant genotypic variation within and between the species (Table 1). Most of the wild genotypes, i.e., GLKS 38153, GLKS 38157, GLKS 38159, GLKS 38161, GLKS 38163, GLKS 38166 (all *chc*), and GLKS 38172 (*S. microdontum* Bitter, abbreviation *mcd*) produced stolons under both HP and LP conditions (Figure 1 and Figure 2). In contrast, only some cultivated genotypes produced stolons (Figure 1 and Figure 2). Stolon biomass ranged from 0.009 g per pot (GLKS 38166, *chc*) to 1.143 g per pot (Amanda) under HP, and from 0.014 g per pot (Paterson’s Victoria) to 0.231 g per pot (GLKS 38166, *chc*) under LP (Figure 2, Table A1).

The R:S ratio showed significant variation among the genotypes (*p* ≤ 0.001), but without significant differences between the treatments (0.231 ± 0.194 for HP and 0.244 ± 0.110 for LP) and species (Table 1 and Table 2). However, the RL per shoot biomass is significantly higher under LP (54.750 cm g^−1^) than under HP (19.200 cm g^−1^), with the native accessions (*S. stenotomum* Juz. & Bukasov, abbreviation *stn*) showing the highest value (Table 1 and Table A1). Notably, the genotypes Paterson’s Victoria, Kristall, and GLKS 24130 (*stn*) recorded significantly higher R:S ratios than the overall mean under HP (Table 2). Among these, Kristall stood out by maintaining a higher R:S ratio than the grand mean under both HP and LP conditions (Table 2).

**Figure 3:**
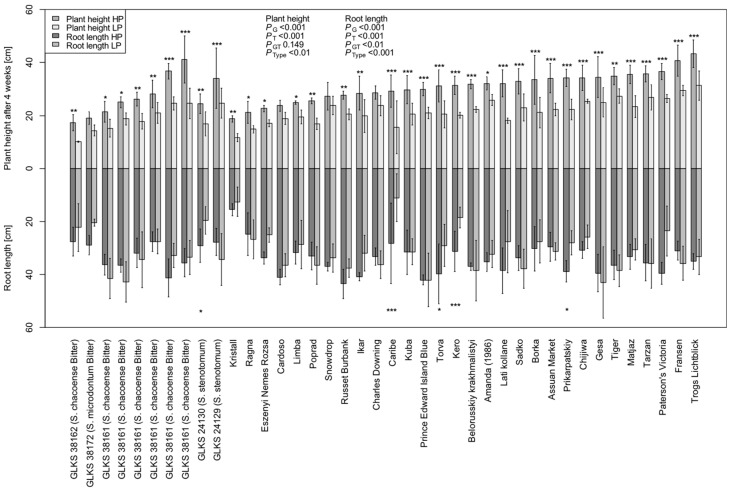
Plant height (PH4) and root length (RL) measured after four weeks under HP and LP conditions; *p*-values represent their ANOVA results for the factors of genotype (G), treatment (T), genotype x treatment (GT), and type/species (Type); asterisks above and below each bar represent significant differences in PH4 and RL between HP and LP within the same genotype, based on the Tukey test at *p* ≤ 0.05 (*), *p* ≤ 0.01 (**) and *p* ≤ 0.001 (***), respectively.

Most of the genotypes showed reduced RL under LP, i.e., 33.825 ± 7.432 cm and 30.875 ± 9.889 cm under HP and LP, respectively (Table A1). However, interestingly, 14 genotypes showed higher RL under LP conditions, namely the varieties Fransen, Belorusskiy krakhmalistyi, Tarzan, Tiger, Gesa, Sadko, Charles Downing, Ragna, Assuan Market, Poprad, and three accessions of *S. chacoense* entries, i.e., GLKS 38153, GLKS 31163, GLKS 36166, along with GLKS 24129 (*stn*) (Figure 3). Plants′ heights measured once a week throughout the duration of the experiment highlighted significant genotypic variation (*p* ≤ 0.001) and significant differences between the treatments (*p* ≤ 0.001), but no G x T effect (Table 1). No significant differences were seen among the various species in the first three weeks (Table 1). However, the cultivated accessions exhibited the highest plant heights towards the end of the experiment (Figure 1), and the difference between the species became more significant after four weeks (*p* ≤ 0.01) (Table A1). The mean PH difference over the four-week period under HP was 25.430 ± 7.102 cm, while it was significantly reduced under LP to an average of 16.837 ± 5.218 cm (Table 1 and Table A1).

CCI values were highest in the cultivated species compared to wild and native accessions under both HP and LP conditions (Figure 1). Overall, both CCI and SPAD values were consistently higher under HP than LP (*p* ≤ 0.001). The mean CCI was 34.504 ± 8.991 in HP and 29.786 ± 9.751 in LP, while SPAD values showed a similar pattern, averaging 46.670 ± 5.021 in HP and 44.016 ± 6.111 in LP (Table A1). Furthermore, both CCI and SPAD exhibited significant genotypic variation (*p* ≤ 0.001) (Table 1).

The P uptake traits (Pupt_shoots, Pupt_roots, and Pupt_total) followed a similar trend, with higher means and greater variability under HP (Figure 1). Total Pupt averaged 5.974 ± 1.853 mg plant^−1^ in HP, whereas it was reduced to 1.479 ± 0.568 mg plant^−1^ in LP, with significant G, T, G x T, and type effects (Table 1 and Table A1). Several cultivated genotypes exhibited significantly higher total Pupt than the grand mean under HP, with the highest value for Belorusskiy krakhmalistyi (8.508 mg plant^−1^), while several others were below the grand mean, including Ragna being the lowest (1.448 mg plant^−1^) (Table 2). Wild accessions generally showed intermediate to low total Pupt under HP. Under LP, total Pupt decreased in all genotypes, with the highest value for Ikar (2.255 mg plant^−1^) and Ranga being the lowest (0.348 mg plant^−1^) (Table A1). Across genotypes, shoot Pupt declined more strongly than root Pupt (−76% vs. −67%) under LP (Table 2).

PuE of the genotypes showed significantly higher values of LP, i.e., 0.227 ± 0.070 mg mg^−1^ under HP and 0.290 ± 0.111 mg mg^−1^ under LP, with cultivated species showing the highest values (Table A1, Table 1, Figure 1). Similarly, PutE was significantly higher under LP conditions (*p* ≤ 0.001), i.e., 0.467± 0.176 mg mg^−1^ under HP and 0.677 ± 0.657 mg mg^−1^ under LP, but with wild accessions showing significantly higher values than the cultivated and native accessions (Table A1, Table 1, Figure 1). Heritability estimates, reflecting the proportion of trait variation attributed to genetic factors, were generally high across all traits, ranging from 57.9% to 95.6% (Table 1). PutE exhibited the highest heritability (95.6%), suggesting predominant control by genetic factors (Table 1). Plant height was also found to be highly genotype specific, with heritability ranging from 86.2% to 92.7% (Table 1). However, traits such as RDW, R:S ratio, and Pupt by shoot and roots displayed moderate heritability, indicating the influence of environmental factors (Table 1).

### 2.2. Hierarchical Clustering of the Genotypes and Traits

**Figure 4:**
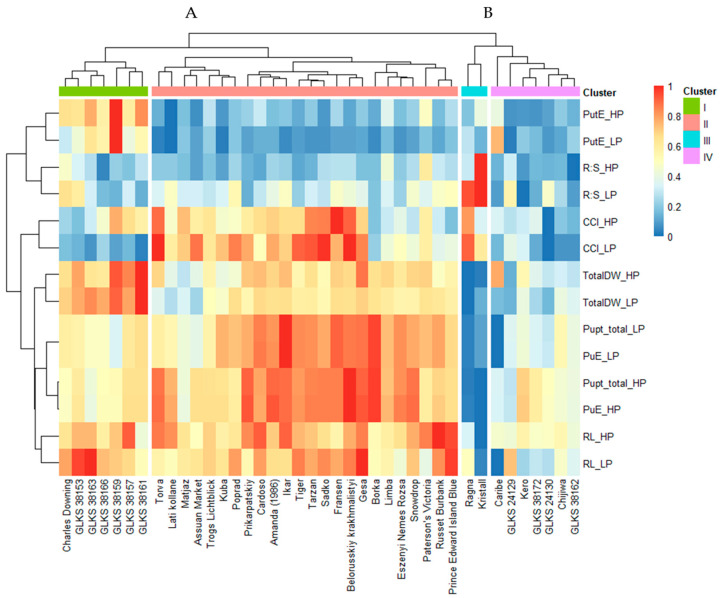
Heatmap based on cluster analysis (distance = Euclidean) of the genotype mean for the phenotypic traits—total dry weight under (TotalDW_HP, TotalDW_LP), root length (RL_HP, RL_LP), root–shoot ratio (R:S_HP, R:S_LP), chlorophyll content index (CCI_HP, CCI_LP), total P uptake (Pupt_total_HP, Pupt_total_LP), P uptake efficiency (PupE_HP, PuE_LP), P utilization efficiency (PutE_HP, PutE_LP) under HP and LP conditions; genotype-specific clusters are represented by the numbers A and B and sub-clusters I, II, III, and IV.

Hierarchical cluster analysis was performed based on the genotype means for six quantitative traits in order to visualize the relationship of the accession phenotypes at high and low P conditions. The heatmap grouped the genotypes into two distinct clusters, i.e., cluster A (31 genotypes) and cluster B (9 genotypes). These were again divided into sub-clusters, one (I) to four (IV). Sub-cluster I contains 7 genotypes, sub-cluster II 24 genotypes, sub-cluster III 2 genotypes, and sub-cluster IV 7 genotypes (Figure 4). Based on the trait means of the genotypes, cluster A can be referred to as a high-performing cluster and cluster B as a low-performing cluster. Sub-cluster I consists of the genotypes with higher biomass (TotalDW), RL, and PutE under HP and LP conditions, but with moderate Pupt under both conditions (Figure 4). Most of the wild accessions belonged to this sub-cluster (*chc*) along with the cultivated variety Charles Downing. Genotypes of sub-cluster II exhibited high Pupt under HP and LP, but low PutE. RL and the biomass accumulation of these genotypes varied between moderate and high, depending on the accession. Most of the cultivated genotypes (Torva, Lati kollane, Matjaz, Assuan Market, Kuba, Poprad, Trogs Lichtblick, Prikarpatskiy, Amanda (1986), Ikar, Tiger, Tarzan, Sadko, Fransen, Belorusskiy krakhmalistyi, and Gesa) in this sub-cluster showed a high CCI value under both HP and LP. However, a few genotypes (Borka, Limba, Eszenyi Nemes Rozsa, Snowdrop, Paterson’s Victoria, Russet Burbank, and Prince Edward Island Blue) showed low values of CCI and moderate biomass accumulation, even though they showed very high PuE under HP and LP. The genotypes Ragna and Kristall, belonging to sub-cluster III, showed the lowest values for most of the traits. But interestingly, both genotypes exhibited a very high R:S ratio under LP conditions. While most of the wild accessions belonged to the sub-cluster I, the two Andean/native accessions (*stn*) belonged to the sub-cluster IV, along with two other wild accessions, i.e., GLKS 38172 (*mcd*) and GLKS 38162 (*chc*). The majority of the genotypes of sub-cluster IV showed moderate to low values for biomass, Pupt, and RL, but a low R:S ratio and PutE under both HP and LP, except for the genotype Caribe, which showed higher PutE under LP, with high biomass accumulation. Regarding the trait clustering, it is evident that the traits related to plants’ growth, like biomass, RL, and CCI, were closely related to PuE, while the PutE was closely related to the R:S ratio.

### 2.3. Correlation Among the Various Phenotypic Traits Under HP and LP

**Figure 5:**
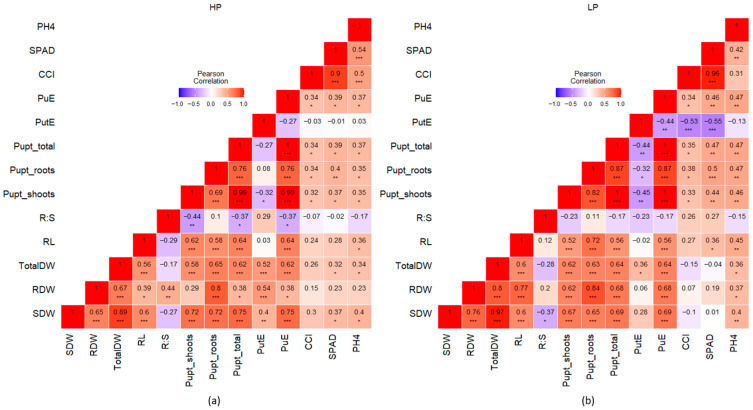
Heatmap of Pearson’s correlation coefficient values of z-score standardized traits under HP (**a**) and LP (**b**); shoot dry weight (SDW), root dry weight (RDW), total dry weight (TotalDW), root length (RL), root−shoot ratio (R:S), P uptake by shoots, roots, and total (Pupt_shoots, Pupt_roots, Pupt_total), P utilization efficiency (PutE), P uptake efficiency (PuE), chlorophyll content index (CCI), Soil Plant Analysis Development (SPAD), and plant height at harvest (PH4); asterisks indicate significant correlations at *p* ≤ 0.05 (*), *p* ≤ 0.01 (**), and *p* ≤ 0.001 (***).

The heatmap of correlation coefficients revealed several significant correlations among the traits and highlighted their variability between the HP and LP conditions (Figure 5). Across both treatments, biomass-related traits—SDW, RDW, TotalDW, and RL—showed positive correlation with P uptake-related traits—Pupt_shoots, Pupt_roots, Pupt_total, PuE, and PutE—except for RL, which showed negative correlation with PutE under LP. However, the strength of their correlation varied between HP and LP (Figure 5).

Under HP, Pupt_total exhibited a stronger significant correlation with SDW (r = 0.75) than RDW (r = 0.38) (Figure 5). A similar pattern can be observed for PuE with SDW and RDW (Figure 5). However, under LP conditions, Pupt_total was almost equally correlated to both RDW (r = 0.68) and SDW (r = 0.69) significantly, instead of a higher correlation with just SDW (Figure 5), as did PuE.

PutE showed a negative correlation with Pupt_shoots (r = −0.32) and Pupt_total (r = −0.27) under HP. However, under the LP condition, PutE was negatively correlated with all the P uptake traits, i.e., Pupt_shoots (r = −0.45), Pupt_roots (r = −0.32), and Pupt_total (r = −0.44) (Figure 5). RL was similarly correlated with Pupt_shoot (r = 0.62) and Pupt_roots (r = 0.58) under HP (Figure 5), whereas under LP, RL showed a stronger correlation to Pupt_roots (r = 0.72) than Pupt_shoots (r = 0.52) (Figure 5). Under LP, PutE showed a negative correlation with PuE (r = −0.44), PH4 (r = −0.13), CCI (r = −0.53), and SPAD (r = −0.55) (Figure 5). A similar trend was observed under HP conditions, except for PH4, which was weakly but positively correlated to PutE (Figure 5). Under both HP and LP, the R:S ratio was negatively correlated to SDW and Pupt_shoots, but showed a higher correlation with RDW under HP (r = 0.44) than LP (r = 0.2) (Figure 5).

## 3. Discussion

### 3.1. Phenotypic Variation of Potato Genotypes in Response to P Deficiency Within and Across Different Species

The screening of 40 potato accessions under HP and LP conditions revealed that the genotypes of cultivated and wild potato species respond differently under P stress conditions. Analysis of variance provided a highly significant variation between treatments for most of the considered traits (Table 1). A decrease in P availability significantly decreased the plant biomass production along with Pupt and PuE, indicating a strong effect of P stress on the genotypes, as had been observed in other crops (wheat [26]; maize [51]; sorghum [52]) (Table A1). As expected, the PutE increased under LP conditions, indicating the plant’s adaptive response to limited P by producing more biomass with fewer resources. In contrast, under HP conditions, plants absorbed more P without fully utilizing it for biomass production, as evident by lower PutE (Table A1). These findings align with previous studies [46,47,53]. Errebhi et al. (1999) screened various wild potato accessions in the field for their response to nitrogen stress and found significant variation among the wild species for their total biomass accumulation [54]. Similarly, in our present study, while the response of most of the traits was genotype specific, quite a high number of the traits were also varying according to different species, like biomass accumulation, P efficiency traits, plant heights, and the leaf chlorophyll contents (Table 1, Figure 1). In plant breeding programs, besides direct traits like yield and biomass-related traits, morphological traits like plant height and CCI under different P treatments could be taken as an indirect trait to select for low P tolerance lines [55]. In our study, the heights of the plants were reduced in parallel to a decrease in P availability (Table A1), confirming previous findings in potatoes as well as other crops [9,56,57]. The rate of this decrease in plant height over the weeks (Diff_PH) varied significantly with genotype and the different species (Table 1), confirming the findings of Lu et al. (2024) [57], where different families of *Gleditsia sinensis* Lam. showed different rates of reduction in their plant heights under low P stress. Interestingly, the plant heights of the wild species were found to be higher than the cultivated ones, indicating their better tolerance and growth under P-limited conditions (Figure 3). Chlorophyll contents, measured as CCI, determine the rate of photosynthesis and play an important role in crops during different stresses to boost up the nutritional status [58,59,60,61,62], and their reduction under P deficiency was found to be associated with ATP reduction, reduced plant biomass, and tuber yield [9]. A study conducted by Kingori et al. (2016) on potatoes found that the CCI could be increased by increasing the fertigation along with an increase in irrigation rate [63]. In our present study, there was a significant reduction in the traits CCI and SPAD under LP conditions, with significant genotypic and species variations (Table A1), indicating a reduction in the rate of photosynthesis as found by other authors, which ultimately explains the reduction in biomass [63,64,65].

Another integral part of a plant’s adaptation to P stress tolerance is the changes in its root system architecture (RSA) during stress conditions [66,67]. Higher P uptake efficiency is usually related to either a larger root system size (higher R:S ratio) or to a higher uptake rate per unit of root length [68,69]. Considerable research on RSA has been conducted in cereals (Lynch 2021), but very little on potatoes [70]. Wishart et al. (2013) demonstrated variability among the *Solanum* groups *tuberosum* and *phureja* regarding their root traits under water and mineral shortage stress under field and greenhouse conditions [71]. They also found that the *phureja* group produced more stolon roots than the *tuberosum* genotypes under stressed greenhouse conditions. Our study also revealed a high variation in root length between different *Solanum* species (Figure 2). Even though most of the genotypes showed reduced root growth under low P availability, several genotypes, including *S. tuberosum*, *S. chacoense*, and *S. stenotomum* species, increased their root length, indicating both the evolutionary adaptation of wild species to low-nutrient environments through changes in root morphology and the genetic potential within cultivated varieties to perform well under nutrient stress. Earlier studies have revealed that horizontal spreading of the stolon roots is important in nutrient acquisition and tuber formation [71,72]. Thus, the production of stolons by the wild *Solanum* species, along with a few cultivated species in our panel, indicates their ability to positively adapt to a P-stress environment for better P acquisition from the soil. A higher R:S ratio is often reported under P-stressed conditions [73]. In our study, there was significant variation among the genotypes regarding their R:S ratio (Table 1). A number of genotypes exhibited a higher R:S ratio under LP conditions (Table 2), indicating a reallocation of energy to roots rather than above-ground biomass under stress conditions, as indicated by a number of other studies [47,48]. However, no significant differences regarding the R:S ratio among the species nor between the treatments were seen in the present study (Table 1). Similar results could be seen in some previous studies performed by Lopez et al. (2023), where no statistical difference in the R:S ratio was found in normalized data between P-deficient and non-deficient treatments [74].

P deficiency in field crops typically results in reduced absolute root length, root length density (RLD), and root biomass, while simultaneously increasing root length relative to shoot biomass [74,75,76]. Our results align with this pattern: although root biomass declined under P stress, root length increased in proportion to shoot biomass. This response supports previous findings that plants adapt to nutrient limitations by reallocating resources to enhance their root system, thereby improving soil exploration and nutrient acquisition. In terms of heritability, our study revealed that several traits, including above-shoot biomass, PutE, PuE, and rate of plant growth, were associated with high broad-sense heritability (Table 1), suggesting strong genetic control and potential in their inclusion as selection indices for P-tolerant genotypes.

### 3.2. Identification of P Tolerant Genotypes

Based on our study, we were able to identify high, moderate, and low P-efficient genotypes (Figure 4). The efficient genotypes revealed strong adaptability traits under stressed conditions, like maintaining good biomass, root growth, and P uptake efficiency under LP (Figure 4). A study conducted on the characterization of *S. chacoense* and *S. tuberosum* by Christensen et al. (2017) showed that all the root traits in *S. chacoense,* including root length, root surface area, and root volume density, were significantly higher than those of the group *S. tuberosum* sub-groups *phureja* and *stenotomum* [77]. In another study conducted by Bachmann-Pfabe and Dehmer (2020), *S. chacoense* accessions were identified as superior in terms of higher nutrient uptake efficiency and tuber starch content under low N conditions [78]. Similar to previous findings, our study revealed that the *S. chacoense* accessions GLKS 38153, GLKS 38163, GLKS 38159, and GLKS 38157 can be categorized as tolerant genotypes due to their enhanced root length and higher biomass production under both HP and LP compared to the rest of the screening panel (Figure 4). Moreover, GLKS 38159 (*chc*) showed exceptional P utilization efficiency (PutE), maintaining high biomass production under both high and low P conditions despite low P uptake (Figure 4). This suggests an inherent ability to use absorbed P more effectively, possibly through enhanced internal P remobilization and allocation to growing tissues [79]. Additionally, alterations in root morphology and physiology, such as increased root surface area or R:S ratio, may contribute to efficient P acquisition and utilization [67]. However, genotype GLKS 38159 (*chc*) appears to defy this pattern as it maintains low root investment but still achieves high P utilization efficiency and biomass, as evident by its low R:S ratio (Table 2). Similarly, we were able to identify a number of outstanding *S. tuberosum* genotypes showing high P acquisition under low P availability (Figure 4). The *S. tuberosum* genotypes—namely Ikar, Tiger, Tarzan, Borka, and Fransen—exhibited high P acquisition under LP (high Pupt_total_LP), indicating their better ability to access soil P even when availability is limited. Most of these genotypes also developed longer root systems under LP (Figure 4), a common adaptive trait that enhances soil exploration and P uptake [80]. The findings regarding the genotype Fransen were consistent with the findings by Wacker-Fester et al. (2019), as they also identified this genotype as tolerant to P stress with high P uptake under both HP and LP conditions [47]. However, despite their high P uptake, these genotypes only achieved medium above-ground biomass with low PutE, suggesting that more biomass was not necessarily translated from high P uptake. These findings highlight that effective P acquisition under stress is not always coupled with high biomass production, pointing to the need to consider both acquisition and utilization traits in breeding programs [81]. Furthermore, the *S. tuberosum* varieties Russet Burbank, Gesa, and Prince Edward Island Blue exhibited exceptionally long root systems under both LP and LP conditions, a key trait associated with enhanced P acquisition through increased soil exploration [82]. Gesa, in particular, combined this with high P uptake efficiency, supporting the idea that genotypic variation in root morphology contributes significantly to nutrient acquisition under variable P availability [83].

Therefore, considering these results, we recommend the introgression of the high P utilization efficiency traits of *S. chacoense* accession GLKS 38159 with the strong P uptake efficiency found in *S. tuberosum* varieties such as Gesa, Ikar, and Fransen in order to develop new cultivars with comprehensive P efficiency.

### 3.3. Correlation Among Various Traits Varies Under High and Low P Availability

The correlation among the various traits revealed that even though the overall picture remained the same, there were notable shifts in the strength of correlation depending on P availability. This reflects the plasticity of plant responses to nutrient availability concerning its resource allocation and stress adaptation (Figure 5) and confirms the findings of Li et al. (2021) regarding the response of maize accessions to low P conditions, where the authors noticed changes in trait correlation patterns under normal and low P conditions [55]. Biomass-related traits exhibited strong positive correlations with P uptake traits, supporting the idea that biomass accumulation is tightly linked to efficient P acquisition (Figure 5), a previously well-established fact. However, the shift from a weaker to a higher correlation of root biomass in comparison to shoot biomass with Pupt under LP conditions highlights a strategic allocation of resources toward root development. This was confirmed with the increase in the RL in some genotypes under LP conditions (Figure 3). Under low P conditions, the root system was the most visibly affected part of the plant [84]. In our study, this shift indicates that plants were employing root-related adaptive strategies to enhance P acquisition, highlighting the critical importance of root traits under low P availability, as has been reported in other crops [70,85]. The negative correlation of PutE with Pupt_shoots and Pupt_total under HP, and even a stronger negative correlation under LP, suggests that higher uptake does not always translate into higher utilization efficiency. This aligns with the concept that plants with high P uptake may not necessarily use P efficiently, especially under limited availability, where metabolic trade-offs may dominate [86]. Although Pupt had a significant effect on biomass production, PutE seems not to have a strong effect on dry matter production. Even though PutE increased under LP in the present study, it was not significantly correlated with dry matter in LP. The only positive significant correlation was observed between PutE and TotalDW under both HP and LP (Figure 5), indicating that higher biomass producers tend to have lower P uptake, which aligns with the findings of Wacker-Fester et al. (2019) [47].

## 4. Materials and Methods

### 4.1. Plant Material and Experimental Design

In the present study, a set of 40 potato accessions from the Gross Luesewitz Potato Collections (GLKS, Gross Luesewitz, Germany) of the Leibniz Institute of Plant Genetics and Crop Plant Research (IPK) was screened for their P efficiency under high and low P conditions (Table S1). The set comprises thirty cultivated accessions of *S. tuberosum* L., seven wild accessions of the species *S. chacoense*, one wild accession of *S. microdontum,* and two native Andean accessions of *S. stenotomum* (Table S1). The cultivated accessions belonged to maturity groups from very early maturity to very late maturity and were released between 1863 and 2018 (Table S1). Detailed passport data of the potato accessions maintained at the IPK Potato Collections in Gross Luesewitz can also be accessed via the genebank information system (GBIS, https://gbis.ipk-gatersleben.de/gbis2i, accessed on 1 June 2025).

The cultivated accessions were selected based on their varying P efficiency as recorded in previous experiments, while the wild accessions were selected based on a previous study on nitrogen efficiency by Bachmann-Pfabe and Dehmer. (2020), which reported that wild potato accessions exhibited superior nitrogen utilization efficiency compared to cultivar varieties [78].

The plant material was maintained in an in vitro (iv) climate chamber at 20 °C. Prior to their cultivation for the experiment, the iv plantlets were tested for diseases like viruses (X, Y, L), potato spindle tuber viroid (PSTVd), and quarantine bacteria (*Clavibacter michiganensis* subsp. *sepedonicus*, *Ralstonia solanacearum*).

The experiment was conducted under controlled greenhouse conditions of 20 °C and 12 h additional light when natural light was below 6 klux. Iv plantlets previously established and maintained on solid Murashige and Skoog (MS) medium [87] at the GLKS, IPK, were used as the starting plant material. Subculturing was performed using both tip and stem cuttings. For tip cuttings, the apical portion of the plantlet, including 2–3 leaves and 1–2 nodes (depending on the plant’s length), was used. Stem cuttings were prepared by cutting a middle portion of the stem containing one node and one leaf. The excised segments were transferred to separate test tubes with sterile MS media. The test tubes were placed in a climate-controlled growth chamber set at 20 °C and maintained for a period of four weeks. These four-week-old iv plantlets of tip and stem cuttings were planted in pots filled with 3.75 kg of high-purity quartz sand substrate (Wolff & Müller Quarzsande GmbH, Röderland, Germany), which was a mixture of various-sized fractions of sand (1:1:1 mixture of the fractions 0.4–0.8, 0.71–1.25, and 1.2–2.5 mm). One plantlet per pot was planted in pots fully saturated with deionized water prior to planting. The experiment was set in a randomized block design with four replications. The plants were irrigated every second day with 100 mL of a modified Hoagland solution [88] comprising high P (HP, 100%) with 15 mg P as KH_2_PO_4_ L^−1^ and low P (LP, 20%) with 3 mg P as KH_2_PO_4_ L^−1^. Both treatments were supplemented with 209.94 mg N as KNO_3_, 214.83 mg K as K_2_SO_4_, 48.36 mg Mg as MgSO_4_7H_2_O, 64.45 mg S as ZnSO_4_7H_2_0, and 200.26 mg Ca as Ca (NO_3_)_2_4H_2_O per L of the nutrient solution. Additionally, micronutrients were added to both treatments in the following amounts per L of nutrient solution: 0.500 mg B as H_3_BO_3_, 0.502 mg Mn as MnSO_4_2H_2_O, 0.050 mg Zn as ZnSO_4_7H_2_O, and 0.012 mg Cu as CuSO_4_5H_2_O and 0.012 mg Mo along with 0.013 mg Na as Na_2_MoO_4_2H_2_0. The pH of the nutrient solutions was maintained at approximately 5.8. The amount of total nutrients added throughout the experiment is listed in Table 3.

### 4.2. Evaluation of Plant Parameters

During the experiment, plant height was measured once a week for four weeks (Table 4). The plants were harvested after 30 days of cultivation. Shoot (SFW), root (RFW), stolon (StolonFW), and tuber (TuberFW) (if produced) biomasses were harvested separately, and their fresh weights were recorded. Root length (RL) was measured for each plant using a ruler during harvesting. After harvesting, the fresh biomass was dried at 60 °C for 3 days, and their dry weights (DW) were measured. The R:S ratio was calculated by dividing the root dry matter by the shoot dry matter.

### 4.3. Determination of Leaf Chlorophyll Content Index (CCI) and Soil–Plant Analysis Development (SPAD)

On the day of harvesting, chlorophyll content of the youngest leaves was measured in terms of chlorophyll content index (CCI) and Soil Plant Analysis Development (SPAD) using Apogee MC-100 (Apogee Instruments, Logan, UT, USA), to assess the relative chlorophyll levels across the genotypes under HP and LP.

### 4.4. Determination of Plant P Content

Dried plant samples were milled and dried again at 105 °C for 2 h, followed by incinerating them at 550 °C for 4–5 h in a muffle furnace. Total P was extracted in 25% HCL [89], and the corresponding P concentrations were measured using inductively coupled plasma-atomic emission spectroscopy (ICP OES Optima 8300, Perkin Elmer, Waltham, MA, USA) at 214 nm wavelength. P uptake (Pupt; mg plant^−1^) of the samples was calculated by multiplying the P concentration by the respective dry weights of the samples. P uptake efficiency (PupE) for the accessions was calculated by dividing the total P taken up (Pupt_total) by the total P applied in the respective treatment

$$\mathrm{PuE}\left(\%\right)=\frac{\mathrm{total}\;P\;\mathrm{uptake}\;(\mathrm{mg}\;\mathrm{per}\;\mathrm{pot})}{\mathrm{total}\;P\;\mathrm{applied}\;(\mathrm{mg}\;\mathrm{per}\;\mathrm{pot})}$$

Furthermore, P utilization efficiency (PutE; mg mg^−1^), indicating the amount of biomass produced per unit of P taken up, was calculated as follows:

$$\mathrm{PutE}\left(\%\right)=\frac{\mathrm{total}\;\mathrm{dry}\;\mathrm{weight}\;(\mathrm{mg}\;\mathrm{per}\;\mathrm{pot})}{\mathrm{total}\;P\;\mathrm{uptake}\;(\mathrm{mg}\;\mathrm{per}\;\mathrm{pot})}$$

### 4.5. Statistical Analysis

All statistical analyses were performed using RStudio (version R-4.2.2) [90]. The data was checked visually for normal distribution of residuals and homogeneity using boxplots and q-q plots.

Analysis of variance (ANOVA) was conducted using linear mixed models fitted with the lmer () function from the “lme4” package [91]. Two models were tested: Model 1 included the fixed effects of genotype (G), treatment (T), and their interaction (G × T), with block as a random effect, while model 2 included the fixed effects of type and type (WKS/AKS/KKS) x treatment (type x T) interaction, with block as a random effect.

A post hoc comparison to the grand mean was carried out for both treatments separately for each trait, thereby comparing the mean of each genotype to the grand mean of the respective treatment. Post hoc pairwise comparisons among all genotype-treatment combinations were conducted using estimated marginal means (emmeans) with Tukey’s adjustment to control for multiple testing. The “emmeans” package was used to calculate contrasts, thereby comparing the mean of each genotype for the respective treatments [92].

Heritability of all the traits was determined using the package “inti” [93]. Pearson’s correlation coefficients and *p*-values between all the traits under HP and LP were calculated using the “Hmisc” package [94]. In order to visualize trait patterns and to identify genotype groupings under HP and LP conditions, a heatmap with hierarchical clustering based on Euclidean distance of selected phenotypic traits was performed using the “pheatmap package” [95]: total dry weight under (TotalDW_HP, TotalDW_LP), root length (RL_HP, RL_LP), root–shoot ratio (R:S_HP, R:S_LP), chlorophyll content index (CCI_HP, CCI_LP), total P uptake (Pupt_total_HP, Pupt_total_LP), P uptake efficiency (PupE_HP, PuE_LP), P utilization efficiency (PutE_HP, PutE_LP).

**Table 4:** List of traits measured in the pot experiment with cultivated and wild potato genotypes, as well as their units and descriptions.

Trait	Abbreviation	Unit	Description
Shoot fresh weight	SFW	g	Fresh weight of the harvested shoot biomass
Root fresh weight	RFW	g	Fresh weight of the harvested root biomass (after washing to remove the sand particles and dry patting)
Shoot dry weight	SDW	g	Weight of the dried shoot biomass (60 °C)
Root dry weight	RDW	g	Weight of the dried root biomass (60 °C)
Tuber fresh weight	TuberFW	g	Fresh weight of the harvested tubers
Tuber dry weight	TuberDW	g	Weight of the dried tubers (60 °C)
Stolon fresh weight	StolonFW	g	Fresh weight of the harvested stolons
Stolon dry weight	StolonDW	g	Weight of dried stolons (60 °C)
Total fresh weight	TotalFW	g	Total weight of the fresh plant biomass as a sum of SFW, RFW, TuberFW, and stolonFW
Total dry weight	TotalDW	g	Total weight of the dried plant biomass as a sum of SDW, RDW, TuberDW, and TuberDW
Root length	RL	cm	Length of the longest root from the base of the shoot
Root–shoot ratio	R:S	-	Root dry weight divided by shoot dry weight
Root length per shoot biomass	RL: SDW	cm g^−1^	Root length divided by shoot dry weight
Plant height after 1 week of planting	PH1	cm	Height of the plant measured up to the top of the newest leaf
Plant height after 2 weeks of planting	PH2	cm	Height of the plant measured up to the top of the newest leaf
Plant height after 3 weeks of planting	PH3	cm	Height of the plant measured up to the top of the newest leaf
Plant height at harvesting, i.e., 4 weeks after planting		cm	Height of the plant measured up to the top of the newest leaf
Difference in plant heights at the beginning and end of the experiment	Diff_PH	cm	Difference between PH1 and PH4
Chlorophyll content index	CCI	-	Chlorophyll content measured at the youngest leaf
Soil–Plant Analysis Development	SPAD	-	Chlorophyll content measured at the youngest leaf
Phosphorus concentration	P_conc	mg (100 g) ^−1^	Amount of P present in the shoot biomass
P uptake	Pupt	mg plant^−1^	Amount of P taken up by the plant
P utilization efficiency	PutE	mg mg^−1^	Amount of biomass produced per unit of P taken up
P uptake efficiency	PuE	mg mg^−1^	Amount of P taken up per unit of P added

## 5. Conclusions

This study provides a comprehensive evaluation of P efficiency across a potato panel of 40 genebank accessions, encompassing cultivated varieties (30) and wild relatives (10) under high and low P conditions. Our findings reveal substantial genotypic and interspecific variation in morphological, physiological, and nutrient uptake traits, highlighting the critical role of genetic background in shaping adaptive responses to P deficiency. We identify specific cultivated and wild potato genotypes with superior P efficiency traits. Wild *S. chacoense* accessions, particularly GLKS 38159, exemplified a high internal P utilization efficiency (PutE), sustaining biomass production under LP despite relatively low P uptake. This strategy, likely driven by efficient internal P remobilization and allocation, represents a highly heritable trait of great value for breeding programs aiming to reduce fertilizer dependency. In contrast, several cultivated genotypes, such as Ikar, Tiger, Tarzan, Borka, and Fransen, displayed enhanced P uptake efficiency (PuE), linked to longer root systems and improved soil exploration. However, this acquisition advantage did not always translate into higher biomass, suggesting trade-offs between uptake and utilization efficiencies. Cluster and correlation analyses confirmed these contrasting strategies, revealing that biomass traits were strongly associated with uptake under both nutrient regimes, while root traits gained greater importance under LP. Thus, the integration of these distinct yet complementary mechanisms—high internal P utilization from wild germplasm and strong P acquisition traits from robust cultivars—can provide a powerful outline for future breeding. Although the study evaluated a limited number of accessions, the contrasting strategies and performance of the accessions exhibit the untapped potential of the wild material. Therefore, these findings strongly support an expanded screening of wild germplasm collections and their intentional inclusion in breeding programs.

## Figures and Tables

**Table 1:** Analysis of variance (ANOVA) and broad-sense heritability (H_bs_^2^) of the phenotypic traits for genotype (G), treatment (T), G x T interaction, and type (cultivated, KKS; wild, WKS; Andean or native sub-collection, AKS) effect.

Trait ^a^	ANOVA ^b^					H_bs_^2^ (%)
	G	T	G x T	Type	Type x T	
SFW	***	***	***	***	N.S.	76.2
SDW	***	***	***	***	N.S.	74.2
RFW	***	***	***	***	N.S.	73.0
RDW	***	***	***	*	N.S.	57.9
R:S	***	N.S.	N.S.	*	N.S.	61.2
RL	***	***	**	N.S.	ns	74.4
RL: SDW	***	***	**	***	*	84.6
TotalFW	***	***	***	***	N.S.	74.8
TotalDW	***	***	***	***	*	71.2
Pupt_shoots	***	***	***	***	*	62.4
Pupt_roots	***	***	***	***	N.S.	61.5
Pupt_total	***	***	***	***	*	62.6
PutE	***	***	N.S.	***	N.S.	95.5
PuE	***	***	**	***	N.S.	88.9
PH1	***	**	N.S.	**	N.S.	86.2
PH2	***	***	N.S.	*	N.S.	89.2
PH3	***	***	N.S.	NS	N.S.	89.3
PH4	***	***	N.S.	***	N.S.	92.6
Diff_PH	***	***	N.S.	***	N.S.	90.7
CCI	***	***	***	***	***	82.3
SPAD	***	***	***	***	***	78.5

^a^ Trait abbreviations are explained in Table 4; ^b^ * *p* ≤ 0.05; ** *p* ≤ 0.01; *** *p* ≤ 0.001; N.S., not significant.

**Table 2:** Root–shoot ratio and phosphorus uptake of genotypes under high-phosphorus (HP) and low-phosphorus (LP) conditions.

Genotype	Species	R:S		Pupt_Shoots [mg/plant]			Pupt_Roots [mg/plant]			Pupt_Total [mg/plant]		
		HP	LP	HP	LP	*p*	HP	LP	*p*	HP	LP	*p*
Eszenyi Nemes Rozsa	*S. tuberosum*	0.210	0.193	**6.375**	1.653	***	0.883	0.265	***	**7.258**	1.918	***
Limba	*S. tuberosum*	0.368	0.236	5.275	1.425	***	**1.235**	0.310	***	6.510	1.735	***
Amanda (1986)	*S. tuberosum*	0.230	0.252	**6.878**	1.568	***	0.888	0.333	***	**7.765**	1.900	***
Fransen	*S. tuberosum*	0.261	0.279	**6.383**	1.718	***	**1.108**	0.355	***	**7.490**	2.073	***
Belorusskiy krakhmalistyi	*S. tuberosum*	0.262	0.263	**7.248**	1.645	***	**1.260**	0.345	***	**8.508**	1.990	***
Chijiwa	*S. tuberosum*	0.183	0.201	3.963	1.135	***	0.540	0.203	**	4.503	1.338	***
Paterson’s Victoria	*S. tuberosum*	0.494 ***	0.238 ***	4.460	1.435	***	**1.133**	0.300	***	5.593	1.735	***
Russet Burbank	*S. tuberosum*	0.277	0.280	5.285	1.478	***	0.930	0.358	***	6.215	1.835	***
Snowdrop	*S. tuberosum*	0.247	0.273	**6.538**	1.470	***	**1.038**	0.350	***	**7.575**	1.820	***
Kuba	*S. tuberosum*	0.138	0.232	5.375	1.523	***	0.598	0.243	**	5.973	1.765	***
Tarzan	*S. tuberosum*	0.171	0.226	**6.500**	1.598	***	0.908	0.320	***	**7.408**	1.918	***
Ikar	*S. tuberosum*	0.217	0.286	**6.745**	1.885	***	0.925	0.370	***	**7.670**	2.255	***
Tiger	*S. tuberosum*	0.147	0.186	**6.363**	1.600	***	0.735	0.323	***	**7.098**	1.923	***
Trogs Lichtblick	*S. tuberosum*	0.200	0.215	5.215	1.158	***	0.765	0.235	***	5.980	1.393	***
Gesa	*S. tuberosum*	0.191	0.244	**6.933**	1.710	***	0.875	0.293	***	**7.808**	2.003	***
Sadko	*S. tuberosum*	0.252	0.212	**6.315**	1.523	***	**1.128**	0.295	***	**7.443**	1.818	***
Borka	*S. tuberosum*	0.186	0.175	**7.363**	1.910	***	0.893	0.248	***	**8.255**	2.158	***
Torva	*S. tuberosum*	0.208	0.235	**6.625**	1.175	***	**0.980**	0.260	***	**7.605**	1.435	***
Charles Downing	*S. tuberosum*	0.388	0.345	3.983	1.135	***	0.850	0.300	***	4.833	1.435	***
Matjaz	*S. tuberosum*	0.186	0.228	3.830	0.945	***	0.498	0.180	*	4.328	1.125	***
Lati kollane	*S. tuberosum*	0.212	0.285	**5.915**	1.165	***	0.828	0.203	***	6.743	1.368	***
Prince Edward Island Blue	*S. tuberosum*	0.249	0.290	5.195	1.305	***	0.900	0.308	***	6.095	1.613	***
Cardoso	*S. tuberosum*	0.154	0.216	**6.320**	1.663	***	0.630	0.315	*	**6.950**	1.978	***
Ragna	*S. tuberosum*	0.278	0.446	1.155	0.260	ns	0.333	0.088	*	1.488	0.348	*
Kero	*S. tuberosum*	0.130	0.110	5.725	1.020	***	0.573	0.110	***	6.298	1.130	***
Assuan Market	*S. tuberosum*	0.139	0.227	5.560	0.858	***	0.543	0.178	**	6.103	1.035	***
Kristall	*S. tuberosum*	0.758 ***	0.471 ***	1.015	0.375	ns	0.265	0.125	ns	1.280	0.500	*
Prikarpatskiy	*S. tuberosum*	0.172	0.159	**6.685**	1.533	***	**1.135**	0.265	***	**7.820**	1.798	***
Poprad	*S. tuberosum*	0.229	0.304	4.658	1.333	***	0.848	0.378	***	5.505	1.710	***
Caribe	*S. tuberosum*	0.175	0.145	3.288	0.243	***	0.463	0.043	***	3.750	0.285	***
GLKS 38153	*S. chacoense* Bitter	0.304	0.323	4.808	1.155	***	0.865	0.248	***	5.673	1.403	***
GLKS 38157	*S. chacoense* Bitter	0.201	0.227	5.183	1.273	***	0.820	0.223	***	6.003	1.495	***
GLKS 38159	*S. chacoense* Bitter	0.221	0.164	4.178	0.765	***	0.858	0.208	***	5.035	0.973	***
GLKS 38161	*S. chacoense* Bitter	0.267	0.158	4.988	1.373	***	**1.013**	0.218	***	6.000	1.590	***
GLKS 38162	*S. chacoense* Bitter	0.071	0.144	4.000	0.933	***	0.303	0.143	ns	4.303	1.075	***
GLKS 38163	*S. chacoense* Bitter	0.251	0.227	3.685	0.943	***	0.653	0.290	**	4.338	1.233	***
GLKS 38166	*S. chacoense* Bitter	0.104	0.170	4.533	1.055	***	0.400	0.193	ns	4.933	1.248	***
GLKS 38172	*S. microdontum* Bitter	0.177	0.157	4.930	0.833	***	0.658	0.123	***	5.588	0.955	***
GLKS 24129	*S. stenotomum* ssp. *stenotomum* Juz. & Bukasov	0.286	0.311	2.963	0.778	***	0.365	0.198	ns	3.328	0.975	***
GLKS 24130	*S. stenotomum* ssp. *stenotomum* Juz. & Bukasov	**0.167**	0.277	4.265	0.715	***	0.583	0.160	***	4.848	0.875	***
Average percent reduction in P uptake under LP				76.0			67.1			75.0		

Bold values indicate genotypes whose mean trait values are significantly higher than the grand mean within each P condition (i.e., separately for HP and LP), based on post hoc comparisons (*p* ≤ 0.05); underlined values indicate genotypes whose mean trait values are significantly lower than the grand mean within each P condition (i.e., separately for HP and LP), based on post hoc comparisons (*p* ≤ 0.05); asterisks denote significant differences between HP and LP within the same genotype for R:S ratio or Pupt (shoots, roots, total) based on Tukey’s test at *p* ≤ 0.05 (*), *p* ≤ 0.01 (**), and *p* ≤ 0.001 (***); non-significant (ns), respectively.

**Table 3:** Total nutrients supplied to the plants per pot in the high P and low P treatments of the P efficiency experiment.

Chemical	Unit	Total Nutrients Applied (mg 1.7L^−1^)	High P	Low P
KHNO_3_	gL^−1^	N	356.90	356.90
K_2_SO_4_	gL^−1^	P	26.31	5.10
KH_2_PO_4_	gL^−1^	K	365.21	365.21
MgSO_4_·7H_2_O	gL^−1^	Mg	82.22	82.22
ZnSO_4_·7H_2_0	gL^−1^	S	109.58	109.58
Ca (NO_3_)_2_·4H_2_O	gL^−1^	Ca	340.45	340.45
H_3_BO_3_	gL^−1^	B	0.85	0.85
MnSO_4_·2H_2_O	gL^−1^	Mn	0.85	0.85
ZnSO_4_·7H_2_O	gL^−1^	Zn	0.085	0.085
CuSO_4_·5H_2_O	gL^−1^	Cu	0.02	0.02
Na2MoO_4_·2H_2_0	gL^−1^	Mo	0.08	0.08
Na2MoO_4_·2H_2_0	gL^−1^	Na	0.02	0.02
FeSO_4_·7H_2_O	gL^−1^	Fe	4.93	4.93

## Data Availability

The original contributions presented in this study are included in the article/Supplementary Material. Further inquiries can be directed to the corresponding author.

## Supplementary Materials

The following supporting information can be downloaded at: https://www.mdpi.com/article/10.3390/plants14243776/s1. Supplementary Table S1: Microsoft Excel file containing (Sheet 1) the list of accessions and (Sheets 2 and 3) phenotypic trait data for 40 accessions grown under high and low P conditions.

## Appendix A

**Table A1:** Minimum (Min), maximum (Max), mean (Mean), and standard deviation (SD) of the phenotypic traits under high-phosphorus (HP) and low-phosphorus (LP) treatments.

Trait ^a^	HP				LP			
	Min	Max	Mean	SD	Min	Max	Mean	SD
SFW	2.111	35.411	21.119	6.745	0.301	15.461	6.480	2.747
SDW	0.253	4.251	2.131	0.804	0.017	1.435	0.685	0.277
RFW	0.247	13.337	4.448	2.918	0.057	6.137	1.709	1.122
RDW	0.019	1.685	0.480	0.308	0.006	0.362	0.158	0.077
R:S	0.039	1.648	0.231	0.194	0.061	0.684	0.244	0.110
RL	6.000	56.000	33.825	7.432	1.000	57.000	30.875	9.889
RL: SDW	7.10	89.02	19.200	11.543	4.367	411.765	54.750	29.412
TuberFW	0.397	6.027	2.691	1.782	0.277	2.687	0.937	0.737
TuberDW	0.076	2.215	0.621	0.582	0.154	0.312	0.228	0.060
StolonFW	0.070	6.700	1.410	1.478	0.100	1.660	0.581	0.422
StolonDW	0.009	1.143	0.209	0.212	0.014	0.231	0.096	0.061
TotalFW	2.478	47.458	26.352	9.430	0.695	18.198	8.401	3.421
TotalDW	0.272	5.253	2.739	1.127	0.095	1.816	0.876	0.343
Pupt_shoots	0.590	8.000	5.190	1.629	0.048	2.760	1.239	0.492
Pupt_roots	0.090	1.785	0.785	0.344	0.011	0.532	0.249	0.109
Pupt_total	0.680	9.571	5.974	1.853	0.063	3.099	1.479	0.568
PutE	0.195	1.477	0.467	0.176	0.362	7.652	0.677	0.657
PuE	0.026	0.364	0.227	0.070	0.012	0.608	0.290	0.111
PH1	2.000	8.000	4.604	1.299	0.500	9.500	4.248	1.415
PH2	4.000	15.000	8.813	2.220	1.500	13.000	7.796	2.003
PH3	9.000	36.500	20.228	5.102	2.000	22.500	14.389	3.646
PH4	14.500	48.500	30.034	7.391	2.000	36.800	21.085	5.669
Diff_PH	11.500	45.000	25.430	7.102	1.500	31.000	16.837	5.218
CCI	19.000	61.000	34.504	8.991	9.000	50.500	29.786	9.751
SPAD	34.900	58.400	46.670	5.021	32.000	55.600	44.016	6.111

^a^ Trait abbreviations are explained in Table 4.
